# The Recreations of Convalescing Soldiers

**Published:** 1914-10-31

**Authors:** Sylvia Spottiswoode

**Affiliations:** Hon. Superintendent.


### The Recreations of Convalescing Soldiers.

*To the Editor of* The Hospital.

Sir,—May I, through the columns of your valuable Journal, offer my sincere thanks to the large number of donors to "The *Tatler* Games Bureau," and make a further appeal for games? So far I have received over 9,900 games, of which number 5,020 were draughts, dominoes, chess, and playing cards.

These I have distributed among seventy-one hospitals and homes where our gallant men are being cared for, but I am still receiving numerous applications, and I am desirous of supplying the camps in Great Britain with games for the winter evenings. I shall be most grateful for any games, cards, dominoes, draughts, or chess sets that your readers care to send to me at "The *Tatler* Games Bureau," Great New Street, London, E.C. The donor's name and address should be written on the outside of each parcel, and acknowledgment will be made in the *Tatler*.—Yours truly,

Sylvia Spottiswoode,
Hon. Superintendent.

